# Electromyographic and Stabilometric Analysis of the Static and Dynamic “Standing Bird Dog” Exercise

**DOI:** 10.3390/sports11060119

**Published:** 2023-06-16

**Authors:** Raffaele Losavio, Samuele Contemori, Stefano Bartoli, Cristina V. Dieni, Roberto Panichi, Andrea Biscarini

**Affiliations:** 1 Department of Nursing and Physiotherapy, University of Salamanca, 37007 Salamanca, Spain; id00793488@usal.es; 2 Centre for Sensorimotor Performance, School of Human Movement and Nutrition Sciences, The University of Queensland, Brisbane, QLD 4071, Australia; s.contemori@uq.net.au; 3 Department of Medicine and Surgery, University of Perugia, 06132 Perugia, Italy; bartolistefano3@gmail.com (S.B.); roberto.panichi@unipg.it (R.P.); 4 Department of Neurobiology, University of Alabama at Birmingham, Birmingham, AL 35233, USA; cdieni@uabmc.edu

**Keywords:** rehabilitation, biomechanics, low back pain, lumbo–pelvic–hip complex, exercise, EMG

## Abstract

(1) Background: The “bird dog” exercise is considered one of the most effective therapeutic exercises for lumbopelvic rehabilitation and the prevention and treatment of low back pain. The “standing bird dog” (SBD) exercise, executed in a single-leg stance, constitutes a natural and challenging variation in the “bird dog”; nevertheless, this exercise has not yet been investigated. This study provides a stabilometric and electromyographic analysis of the SBD performed in static and dynamic conditions and in ipsilateral and contralateral variations; (2) Methods: A time-synchronized motion capture system, wireless electromyography sensors, and triaxial force platform were used to analyze the selected SBD exercises; (3) Results: In dynamic conditions, the gluteus maximum, multifidus, lumbar erector spinae, and gluteus medius reached a mean activation level higher than in the static condition, with peak activation levels of 80%, 60%, 55%, and a 45% maximum voluntary isometric contraction, respectively. In the static condition, balance control was more challenging in the mediolateral compared to the anteroposterior direction. In the dynamic condition, the balance challenge was higher in the anteroposterior direction and higher than the static condition in both directions; (4) Conclusions: The SBD was proved to be effective for strengthening the hip and lumbar extensor muscles and provided a powerful challenge to single-leg balance control in both mediolateral and anteroposterior directions.

## 1. Introduction

The “bird dog” is one of the most popular core exercises [1,2], is commonly prescribed in lumbopelvic rehabilitation interventions [3], and is considered one of the most effective therapeutic exercises for the prevention and treatment of low back pain [4]. Several electromyographic (EMG) studies have evaluated the activity of core muscles during the bird dog exercise [5,6,7,8,9,10,11,12,13,14,15,16,17,18,19,20]. Overall, these studies provide evidence that this exercise primarily targets the lumbar and hip extensor muscles and reported activation levels of 21–56%, 23–42%, 21–42%, 23–46%, 22–38%, 21–35%, and 4–15% maximum voluntary contractions (MVIC) were obtained for the gluteus maximus, gluteus medius, hamstrings, lumbar multifidus, longissimus thoracis, external oblique abdominis, and rectus abdominis, respectively. Notably, gluteal muscle weakness was associated with poor lumbopelvic posture [21] and with an increased risk of lower limb injuries [22]; weakness and lack of endurance in the lumbar extensor muscles were correlated with low back pain [23,24]. 

Different variations of the bird dog have been devised to render the exercise more challenging, produce higher levels of muscle activations, and develop superior adaptations. For example, this exercise can be executed with external weights at the wrist and ankle [14], using unstable supporting surfaces [11,18] and changing the base of support, including assuming a plank position [12] or a standing position instead of a quadruped one. 

The use of wrist/ankle weights has also led to the higher activation of the lumbar multifidus and erector spinae muscle [14]. External loads at the distal segments, however, also considerably increase the shear components of the reaction forces that act on the joints of the trunk and limbs as these segments are positioned horizontally. These shear forces are indeed proportional to the distance between the joint and the vertical line of action in the gravitational resistance [25,26].

Using unstable surfaces can significantly improve neuromuscular control by promoting anticipatory and reactive postural adjustments and enhancing the sensitivity of afferent feedback pathways [27,28,29]. Overall, compared with the same exercises performed under stable conditions, strength exercises performed on unstable surfaces yield a higher core-muscle activation and perceived exertion but a lower force generation, power output, movement velocity, and range of motion [27,28,29]. Specifically, compared to the ground-based exercise, the dynamic bird dog executed on a whole-body wobble board developed a higher activation of external and internal obliques, the serratus anterior, and trapezius medius, which was nearly the same as the gluteus maximum activation, and lower activation of the multifidus and erector spinae [18]. 

Executing the bird dog exercise from the plank rather than a quadruped position also enhances the activity of the rectus abdominis, transversus abdominis, and external obliques yet reduces that of the multifidus and erector spinae, thus resulting in less effective outcomes for the lumbar musculature [12]. This effect is likely a direct consequence of the “long lever” body position induced by the plank posture. 

The standing bird dog (SBD), i.e., the bird dog exercise executed in a standing body position, demonstrates biomechanical features that classify it as one of the most challenging variations of the traditional quadruped bird dog exercise. In the starting position, the exerciser stands straight with a neutral spine position, knees fully extended, arms straight at the sides of the body, and feet flat on the floor about hip-width apart. From this position, the trunk slowly leans forward, simultaneously raising one leg backward and the arm on the opposite side forward (through simultaneous unilateral kip extension and contralateral shoulder flexion) and keeping the other arm at the side against the body. During the movement, the subject should maintain a neutral spine position, hips at the same level (to avoid pelvic rotations in the transverse plane), and proper knee alignment (to avoid knee varus/valgus and internal/external rotation). In the final position, the supporting leg is nearly perpendicular to the ground, while the trunk, the upper limbs, and the non-supporting leg should be in-line and parallel to the ground, with the trunk facing the ground. In the isometric SBD, this position is held for a selected time before the subject gradually returns to the starting standing position. In the dynamic SBD, this exercise is performed in a continuous motion, alternating the sides of the body during consecutive repetitions or sets. Unlike the abovementioned variations of the bird-dog exercise, the SBD has not yet been investigated in either its isometric or dynamic form.

The aim of this study was to determine the stabilometric parameters and the EMG activity of the gluteal and lumbar extensor muscles during static and dynamic SBD exercises. This single-leg stance was maintained during the SBD, in the absence of contact between the upper limbs and ground and in addition to the hip, which also exposes the spine to a considerable gravitational flexion torque. Thus, we hypothesized that the gluteal and lumbar extensor muscles could reach considerable levels of activity, despite the exercise being performed on a stable support surface and with no external overload. We also hypothesized that the reduced base of support and the challenging SBD body position required a balance control that was considerably higher during standing in a single-leg stance. Furthermore, unlike the quadruped bird dog, the SBD could be easily performed ipsilaterally, i.e., by raising a lower and an upper limb on the same side of the body. Thus, both the contralateral and ipsilateral variations of the SBD were analyzed in this study.

## 2. Materials and Methods

### 2.1. Participants

Five females and fifteen males (mean age: 24 y, range: 21–39 y; mean height: 1.77 m, range: 1.59–1.91 m; and mean body mass: 75 kg, range: 52–90 kg) recruited from local fitness centers participated in the study. Based on preliminary data, the participant sample size was determined by a priori power analysis using the G*Power 3.1.9.7 program (Test family: F-test; Statistical test: ANOVA, repeated measures, within-subjects; Type of power analysis: a priori), with a significance alpha level set at 0.05, a statistical power of 0.8, and a medium-high effect size corresponding to a Cohen’s *d* effect size equal to 0.65. Only subjects with proven experience in bodyweight resistance training (intermediate to advanced level) and specifically with core exercises were considered for inclusion in this study. Exclusion criteria included musculoskeletal injuries, a previous record of limb and trunk pathologies, and the inability to perform the SBD exercise without pain and with proper form and technique. All participants gave informed consent to their inclusion in the study and were free to withdraw at any time during the experiment, which was approved by the ethics committee of the University of Perugia and conducted in accordance with the Declaration of Helsinki.

### 2.2. Selected Exercises and Muscles

**Figure 1:**
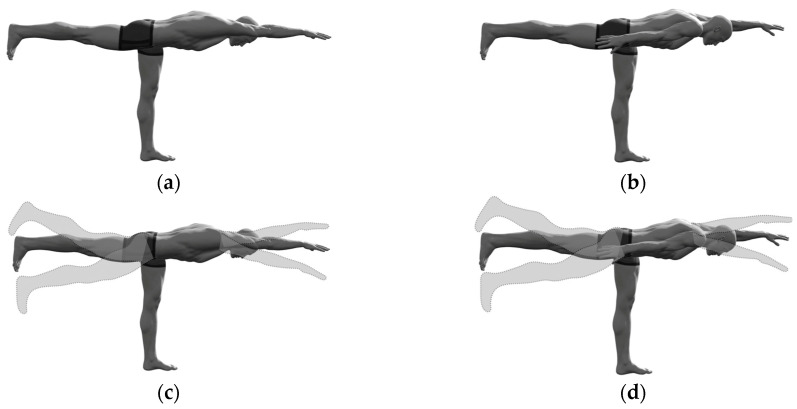
The selected “standing bird dog” exercise variations: (**a**) Static ipsilateral; (**b**) Static contralateral; (**c**) Dynamic ipsilateral; (**b**) Dynamic contralateral.

Each participant performed the contralateral (raising one leg and the opposite-side arm) and ipsilateral (raising one leg and arm on the same side of the body) SBD exercise in isometric and dynamic conditions (Figure 1). Each static position was held for 12 s. In the dynamic condition, each participant executed 12 short-range movements about the static position at a constant cadence, with a 0.5-s concentric phase and a 0.5-s eccentric phase, and with a range of hip and shoulder flexion/extension movements comprised between 10° and 15°. During each trial, EMG signals were recorded bilaterally from the gluteus maximus (GMAX), gluteus medius (GMED), the lumbar part of the erector spinae longissimus (ES), and lumbar multifidus (MF).

### 2.3. Familiarization Session

One week before the testing session, each participant underwent a pretest familiarization session during which the testing protocol was explained in detail, and the exercises were first illustrated by a professional trainer and then practiced by the participants under his supervision. The participants also learned to execute the exercise repetitions at the selected cadence, following the ticks emitted by a metronome set at 1 beat per second, and a selected range of movement, with the aid of tactile feedback. At the end of the session, all the participants were able to perform the exercises with the correct technique. The 12-s duration of the trials was precisely selected by observing that trials lasting longer than 12 s began to be affected by fatigue in some participants.

### 2.4. Testing Session

After a 10 min warm-up on a bike ergometer at self-selected resistance, we recorded the EMG activity during the maximum voluntary isometric contractions (MVIC) of the selected muscles following a procedure previously described by Biscarini et al. [18]. After the MVIC tests and full recovery (of at least 5 min), each participant performed the four selected SBD exercise variations with the foot of the supporting leg resting on a force platform. The trials were randomly intermingled and separated by a 3 min resting phase. The EMG signals recorded during the MVIC tests were used for the offline normalization of the EMG signals recorded during the SBD trials (see the next section for details).

### 2.5. Data Recording and Processing

Surface EMG signals from the eight selected muscles were recorded with wireless EMG sensors (FreeEMG 1000; BTS Bioengineering, Milano, Italy) and Ag/AgCl surface electrodes placed on the muscle belly, parallel to the muscle fibers, 2 cm apart from each other. The precise electrode placement for each muscle was reported in a previous paper [18]. The raw EMG signals were differentially amplified (933 gain), bandpass filtered (10–500 Hz), digitalized (16-bit resolution, 1-kHz sampling frequency), and transformed into amplitude envelopes through a point-to-point moving root mean square filter (500-ms time interval). The EGM amplitude envelopes recorded from each muscle during the SBD trials were then normalized to the maximum of the EGM amplitude envelope obtained from the MVIC test of the same muscle. Finally, we computed the mean value of each normalized EMG signal, and the peak value of the ones relative to the dynamic SBD trials was obtained as the mean of the peak values recorded in each of the 12 repetitions. 

The stabilometric parameters of the supporting leg were obtained via a “BTS P-6000” force platform (BTS Bioengineering, Milano, Italy). The signal recording was accomplished with a 16-bit A/D converter and a 500 Hz sampling frequency. Only the central 10 s of each recording was considered for the analysis of the relevant two-dimensional center of pressure (CoP) parameters (mean velocity and excursion area) and one-dimensional mediolateral and anteroposterior CoP parameters (standard deviation and mean velocity).

Kinematic data recording was conducted to identify the single repetitions during a dynamic trial, thus enabling the determination of the peak EMG values and the range of hip and shoulder movements in each repetition. Specifically, we used an 8-camera optoelectronic motion capture system (Smart-DX 6000; BTS Bioengineering, Milano, Italy) to track the reflective markers that were attached bilaterally to bone landmarks at the wrist, ankle, hip, shoulder, elbow, and wrist joints of the body. A detailed description of the motion capture system can be found in our previous papers [30,31].

The EMG, force plate, and optoelectronic systems were synchronized in time and provided with native Smart Capture^®^ 1.10 software for data acquisition and Smart Analyzer^®^ 1.10 and Sway^®^ 1.4 software for data analysis.

### 2.6. Statistical Analysis

Analysis of variance (ANOVA) was performed to evaluate statistically significant differences between SBD variations. Normality, the variance between populations and sphericity were assayed by the Shapiro–Wilk test, Levene’s test, and Mauchly’s sphericity test, respectively. Probabilities were corrected based on Greenhouse–Geisser, and Huynh–Feldt epsilon when appropriated. Data that did not follow ANOVA assumptions for normality and variances were transformed by applying an ln function to allow transformed data to satisfy the ANOVA assumptions. The mean ± SD used for descriptive statistics was always referred to as the original data, even when the statistical analysis was carried out on the transformed data. 

EMG data samples were analyzed with 3-way repeated measures of ANOVA with ipsilateral/contralateral exercise variations in a static/dynamic condition and the supporting/non-supporting side of the body as independent within-subject factors. These two-dimensional CoP stabilometric parameters were analyzed with 2-way repeated-measures ANOVA with ipsilateral/contralateral exercise variation and a static/dynamic condition as independent within-subject factors; for the one-dimensional CoP parameters, 3-way repeated-measures of ANOVA were used with a mediolateral/anteroposterior direction as the third independent within-subject factor. The comparison between the change in the mediolateral component and the change in the anteroposterior component of a one-dimensional CoP parameter was obtained with the same ANOVA method used for the two-dimensional parameters. For the significant main effects or interactions, the statistical power and effect size were assessed by the observed power (ω) and partial eta squared ($\eta_{p}^{2}$) coefficients, respectively, and post hoc analysis was run via the Scheffè test. The significance level was designated at *p* < 0.05 for each statistical test. Among all the many possible comparisons, we considered only those of interest for this research study. The SPSS software package was used for statistical calculations. 

## 3. Results

### 3.1. Stabilometric Parametrs

**Figure 2:**
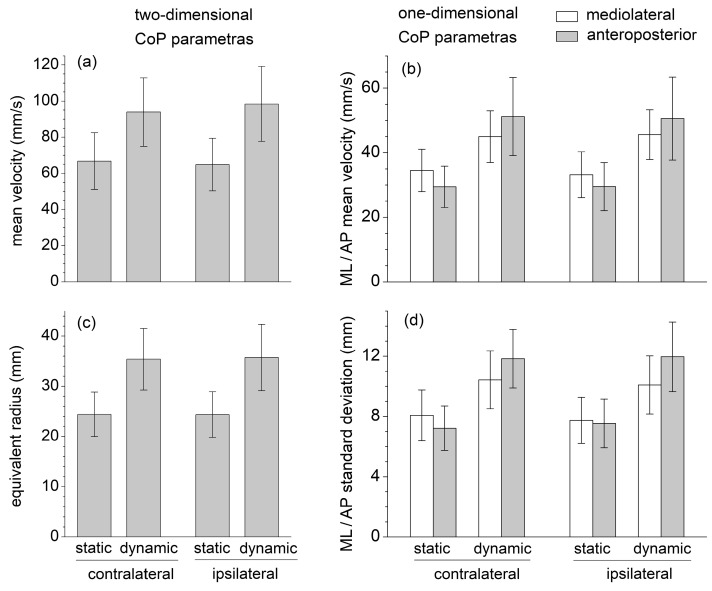
One-dimensional and two-dimensional center of pressure (CoP) parameters recorded during the SBD exercises executed in static and dynamic conditions, and in contralateral and ipsilateral variations: (**a**) Mean velocity; (**b**) Mediolateral and anteroposterior mean velocity; (**c**) Equivalent radius of excursion area; (**d**) Standard deviation of mediolateral and anteroposterior displacement.

None of the six selected CoP displacement parameters (mean velocity, equivalent radius of excursion area, mediolateral and anteroposterior standard deviation, and mean velocity) were significantly affected by the contralateral/ipsilateral exercise variation (Figure 2). The intraclass correlation coefficient ICC was estimated, and their 95% confidence interval (model: test–retest/intra-rater reliability, type: single measurement, definition: absolute agreement) was calculated for each corresponding contralateral/ipsilateral pair of sets of measurements (Table 1). The ICC estimates indicated good reliability for the two-dimensional CoP parameters, moderate reliability for the mediolateral and anteroposterior mean CoP velocity, and poor reliability for the mediolateral and anteroposterior CoP standard deviation [32].

All stabilometric parameters were significantly greater in dynamic than in static conditions (p<0.001, $\eta_{p}^{2}>0.79$, ω=1.00), reflecting a greater body instability during the dynamic executions of the SBD (Figure 1). For these static conditions, one-dimensional mediolateral parameters were greater than the corresponding anteroposterior ones, whereas the opposite results were obtained in the dynamic conditions. Despite some of these differences appearing statistically insignificant, following the transition from the static to the dynamic execution, the increase in all the anteroposterior parameters was always significantly larger than those in the mediolateral ones (p<0.001, $\eta_{p}^{2}\geq 0.55$, ω>0.99).

### 3.2. EMG Parameters

**Figure 3:**
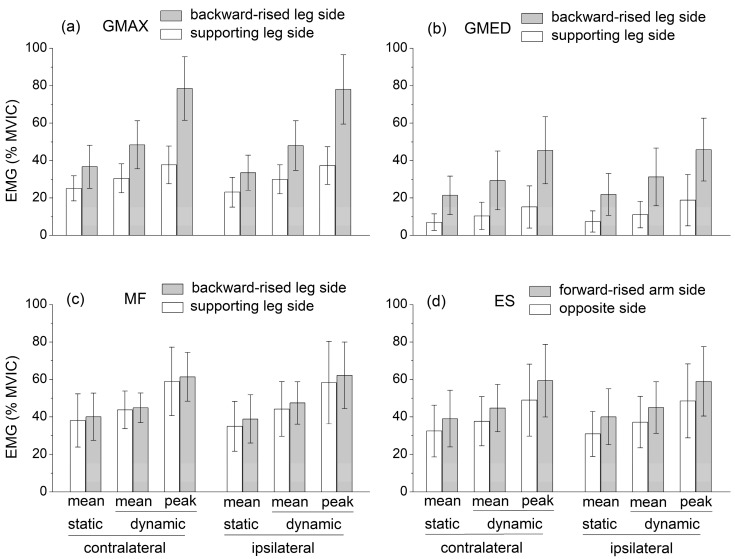
Mean muscle activation levels recorded during the static SBD and mean and peak muscle activation levels, which were recorded during the dynamic SBD: (**a**) Gluteus maximum (GMAX), (**b**) Gluteus medius (GMED), (**c**) Multifidus (MF), (**d**) Lumbar erector spinae (ES). Both the contralateral and ipsilateral exercise variations are displayed. Muscle activation levels are expressed as a percentage of the peak electromyographic amplitude at MVIC.

The independent variables had quite different effects on the mean EMG level of the selected muscles (Figure 3). The ipsilateral/contralateral variant had no significant effect on muscle activation levels. 

The GMAX and GMED activation was significantly higher at the side of the backward-raised lower limb (p<0.001, ω=1.00, $\eta_{p}^{2}=0.69$ for GMAX, $\eta_{p}^{2}=0.72$ for GMED), ES activation was significantly higher at the side of the forward-raised upper limb (p=0.001, $\eta_{p}^{2}=0.47$, ω=0.97), and MF activity was not significantly different between the sides of the body. 

The mean activation of all the muscles was significantly higher in dynamic compared to static conditions (p≤0.001, $0.43\leq \eta_{p}^{2}\leq 0.68$, ω≥0.95). Notably, the peak EMG levels reached by GMAX, MF, ES, and GMED in dynamic conditions were about 80%, 60%, 60%, and 45% MVIC, respectively.

## 4. Discussion

This study provided a stabilometric and electromyographic analysis of the standing bird dog exercise performed in static and dynamic conditions and in contralateral (rising a lower and upper limb at opposite sides of the body) and ipsilateral (rising a lower and upper limb at the same side of the body) variations. We found that SBD was an effective exercise for strengthening the hip and lumbar extensor muscles and provided a powerful challenge to single-leg balance control in both the mediolateral and anteroposterior directions.

EMG data highlight that the mean muscle activation levels of GMAX, GMED, ES, and MF during the static SBD were significantly lower than the corresponding mean values obtained during the dynamic SBD executed with short-range movements about the static position (Figure 3). The values of all the CoP displacement parameters (mean velocity, equivalent radius of excursion area, mediolateral and anteroposterior standard deviation, and mean velocity) were also significantly lower in static than in dynamic conditions (Figure 2). This suggests that the selected dynamic version of the SBD exercise constituted a challenging variation in the static SBD, which required a higher muscle effort and balance control relative to the static condition.

During the static SBD, the CoP standard deviation and mean velocity were higher in the mediolateral than anteroposterior direction, plausibly due to the different dimensions at the base of the support (the foot plant) in the two directions (Figure 2). The sagittal-plane limb movements performed in dynamic conditions yielded a considerable increase (about 70%) in the value of the anteroposterior parameters; nevertheless, it also induced a statistically significant increase (about 25%) in the mediolateral parameters. This result highlighted how the destabilization effects caused by an internal perturbation acting in one direction could be spread in different directions. 

The available EMG levels of the hip and lumbar extensor muscles during the static quadruped bird dog exercise were characterized by a large variability (see Section 1). Nevertheless, when averaged over the existing studies, these values could be compared to those obtained in the present study during the static SBD. Contrary to the GMED, MF, and ES activation levels were higher in the SBD than in the quadruped bird dog exercise. In both the quadruped and the standing bird dog, the activation levels of GMAX and GMED were higher at the side of the raised lower limbs, while the ES activity was higher at the side of the raised upper limb. However, unlike the quadruped bird dog, during the SBD, MF activity was not significantly different between the sides of the body. Notably, in the dynamic SBD, GMAX, MF, and ES reached mean activation levels higher than 40% MVIC, and peak activation levels of about 80%, 60%, and 60% MVIC, respectively, avoiding the use of any overload, unstable surface, or explosive movement (Figure 3). These levels were considerably higher compared to those recorded during other ground-based bodyweight exercises, which were designed for strengthening hip and lumbar extensor muscles (e.g., the supine bridge exercise) and performed by keeping the spine safely in a neutral position [5,6,7,8,9,10,11,12,13,14,15,16,17,18,19,20].

Contrary to the quadruped bird-dog exercise, SBD provides a considerable challenge to balance control. Exercises performed in a single-leg stance or on unstable supporting surfaces could significantly improve neuromuscular control by promoting proactive and reactive postural adjustments and enhancing the sensitivity of afferent feedback pathways [27,28,29]. Postural control and joint stability in the single-leg stance are fundamental components of many natural human movements and sports actions and often constitute a critical factor in lower limb athletic rehabilitation and injury prevention during contact sports. Notably, compared to quiet standing in a single-leg stance [33], the CoP velocity and excursion area are about two and six times higher, respectively, during the static SBD; and three and nine times higher during the dynamic SBD (Figure 2). 

In the dynamic SBD trials, hip and shoulder flexion/extension movements were executed with a short range (10–15°) about the static position. Instead, in the standard SBD exercise, a full ROM is typically used, from quiet standing in a single-leg stance to the position maintained during the static trial. The hip and shoulder ROM was limited to maximize the mean muscle activity during repetition, enabling a more effective exercise and for a meaningful comparison with the static trials and the quadruped bird dog. In fact, while reaching the standing reference position, the hip and shoulder flexion/extensor external torque developed by gravity was gradually reduced to negligible values, and the activation level of at least the GMAX and GMED of the non-supporting leg was expected to become insignificant. 

Ultimately, this study highlighted that SBD is an effective exercise for strengthening the hip and lumbar extensor muscles and provides a powerful challenge to single-leg balance control. Additionally, it can be considered a safe exercise as it is performed with the spine in a neutral position and without the use of external overloads and unstable surfaces. However, correct exercise execution requires considerable levels of flexibility, balance control, and joint stability. Therefore, SBD is more suitable for individuals experienced in resistance training, athletic training regimens, injury prevention in contact sports, and during the late functional phase of athletic rehabilitation programs.

The limitations of this study are mainly related to the biomechanical features of the SBD exercise. Specifically, the CoP position was recorded during a 12-s time interval, which is considerably shorter than that recommended for standard postural assessments executed in the standing position. However, compared to standing in a double-leg or single-leg stance, the selected SBD exercises were considerably more challenging, and trials lasting longer than 12 s could likely have been made biased by fatigue. The stabilometric parameters subsequently recorded in ipsilateral and contralateral exercise conditions, which displayed no statistically significant difference, were used to assess data test–retest reliability through the calculation of the ICC coefficients. The results suggested good reliability (0.75 < ICC < 0.9) [32], at least for the two-dimensional CoP parameters. 

During the dynamic SBD trials, participants were not given feedback on their hip and shoulder range of motion and repetition cadence. This feedback was only provided in the familiarization session until the experimenters and professional supervisor trainers judged the exercise execution to be satisfactory. During the trials, participants were only asked to reproduce the exercise execution focusing on balance and proper exercise form. In fact, a sharp focus on cadence and range of motion could have a negative impact on balance, joint alignment, and correct body position. Only a few trials that demonstrated a mean value of repetition cadence and range of motion (determined by motion analysis) that differed more than 20% from the selected values (1 s cadence, 10–15° range of motion) were repeated by participants.

The hip and lumbar extensors were the target muscles of the SBD exercise, as they primarily oppose the hip and spine flexor torque and are induced by gravity. However, other muscles, particularly the internal and external obliques and the tibialis posterior and the fibularis muscles could contribute to body stabilization due to the challenging asymmetric body position maintained in a single-leg stance. Therefore, further studies are needed to achieve a full EMG characterization of the SBD exercise. 

## 5. Conclusions

This study provided a stabilometric and electromyographic analysis of different variations of the SBD exercise. These results highlight that static and dynamic SBD can be included sequentially in the final stage of exercise progressions that gradually challenge the lumbar extensor muscles and single-leg balance control (in both the mediolateral and anteroposterior directions), avoiding the use of external overloads and unstable supporting surfaces.

## Figures and Tables

**Table 1:** Interclass correlation coefficient (model: test–retest/intra-rater reliability, type: single measurement, definition: absolute agreement) of the CoP stabilometric parameters for each corresponding contralateral/ipsilateral pair of sets of measurements.

CoP Parameter	Condition	Interclass Correlation	95% Confidence Interval	
			Lower Bound	Upper Bound
Velocity	Static	0.878	0.723	0.950
	Dynamic	0.798	0.558	0.915
Equivalent radius	Static	0.798	0.555	0.915
	Dynamic	0.812	0.584	0.921
ML velocity	Static	0.590	0.219	0.814
	Dynamic	0.786	0.536	0.910
AP velocity	Static	0.726	0.423	0.882
	Dynamic	0.638	0.277	0.840
ML standard deviation	Static	0.281	−0.177	0.637
	Dynamic	0.756	0.488	0.895
AP standard deviation	Static	0.431	0.000	0.727
	Dynamic	0.455	0.014	0,774

## Data Availability

The data that support the findings of this study are available from the corresponding author upon reasonable request.

## References

[1] McGill S.M. (1998). Low back exercises: Evidence for improving exercise regimens. Phys. Ther..

[2] Huxel Bliven K.C., Anderson B.E. (2013). Core stability training for injury prevention. Sport. Health.

[3] Kisner C., Colby L. (2012). Therapeutic Exercise: Foundations and Techniques.

[4] McGill S.M. (2017). Ultimate Back Fitness and Performance.

[5] Konrad P., Schmitz K., Denner A. (2001). Neuromuscular Evaluation of Trunk-Training Exercises. J. Athl. Train.

[6] Marshall P.W., Murphy B.A. (2005). Core stability exercises on and off a Swiss ball. Arch. Phys. Med. Rehabil..

[7] Drake J.D., Fischer S.L., Brown S.H., Callaghan J.P. (2006). Do exercise balls provide a training advantage for trunk extensor exercises? A biomechanical evaluation. J. Manip. Physiol. Ther..

[8] Stevens V.K., Vleeming A., Bouche K.G., Mahieu N.N., Vanderstraeten G.G., Danneels L.A. (2007). Electromyographic activity of trunk and hip muscles during stabilization exercises in four-point kneeling in healthy volunteers. Eur. Spine J..

[9] Ekstrom R.A., Donatelli R.A., Carp K.C. (2007). Electromyographic analysis of core trunk, hip, and thigh muscles during 9 rehabilitation exercises. J. Orthop. Sport. Phys. Ther..

[10] Ekstrom R.A., Osborn R.W., Hauer P.L. (2008). Surface electromyographic analysis of the low back muscles during rehabilitation exercises. J. Orthop. Sport. Phys. Ther..

[11] Imai A., Kaneoka K., Okubo Y., Shiina I., Tatsumura M., Izumi S., Shiraki H. (2010). Trunk muscle activity during lumbar stabilization exercises on both a stable and unstable surface. J. Orthop. Sport. Phys. Ther..

[12] Okubo Y., Kaneoka K., Imai A., Shiina I., Tatsumura M., Izumi S., Miyakawa S. (2010). Electromyographic analysis of transversus abdominis and lumbar multifidus using wire electrodes during lumbar stabilization exercises. J. Orthop. Sport. Phys. Ther..

[13] Guo L.Y., Wang Y.L., Huang Y.H., Yang C.H., Hou Y.Y., Harn H.I., You Y.L. (2012). Comparison of the electromyographic activation level and unilateral selectivity of erector spinae during different selected movements. Int. J. Rehabil. Res..

[14] Masaki M., Tateuchi H., Tsukagoshi R., Ibuki S., Ichihashi N. (2015). Electromyographic analysis of training to selectively strengthen the lumbar multifidus muscle: Effects of different lifting directions and weight loading of the extremities during quadruped upper and lower extremity lifts. J. Manip. Physiol. Ther..

[15] Yoon T.L., Cynn H.S., Choi S.A., Choi W.J., Jeong H.J., Lee J.H., Choi B.S. (2015). Trunk muscle activation during different quadruped stabilization exercises in individuals with chronic low back pain. Physiother. Res. Int..

[16] Kelly M., Jacobs D., Wooten M.E., Edeer A.O. (2016). Comparison of electromyographic activities of lumbar iliocostalis and lumbar multifidus muscles during stabilization exercises in prone, quadruped, and sitting positions. J. Phys. Ther. Sci..

[17] Kim C.R., Park D.K., Lee S.T., Ryu J.S. (2016). Electromyographic Changes in Trunk Muscles during Graded Lumbar Stabilization Exercises. PMR.

[18] Biscarini A., Contemori S., Grolla G. (2019). Activation of Scapular and Lumbopelvic Muscles During Core Exercises Executed on a Whole-Body Wobble Board. J. Sport Rehabil..

[19] Lehecka B.J., Stoffregen S., May A., Thomas J., Mettling A., Hoover J., Hafenstine R., Hakansson N.A. (2021). Gluteal Muscle Activation During Common Yoga Poses. Int. J. Sport. Phys. Ther..

[20] Park S., Lim W. (2022). Activity of Posterior Oblique Sling Muscles during Quadruped Hip Extension with Different Shoulder Positions. Int. J. Hum. Mov. Sport. Sci..

[21] Neumann D.A. (2010). Kinesiology of the Musculoskeletal System.

[22] Distefano L.J., Blackburn J.T., Marshall S.W., Padua D.A. (2009). Gluteal muscle activation during common therapeutic exercises. J. Orthop. Sport. Phys. Ther..

[23] Hultman G., Nordin M., Saraste H., Ohlsén H. (1993). Body composition, endurance, strength, cross-sectional area, and density of mm erector spinae in men with and without low back pain. J. Spinal Disord..

[24] Nourbakhsh M.R., Arab A.M. (2002). Relationship between mechanical factors and incidence of low back pain. J. Orthop. Sport. Phys. Ther..

[25] Biscarini A., Busti D., Calandra A., Contemori S. (2017). The “supine bridge” therapeutic exercise: Determination of joint torques by means of biomechanical modeling and technologies. J. Mech. Med. Biol..

[26] Biscarini A. (2021). Non-Slender n-Link Chain Driven by Single-Joint and Multi-Joint Muscle Actuators: Closed-Form Dynamic Equations and Joint Reaction Forces. Appl. Sci..

[27] Behm D.G., Drinkwater E.J., Willardson J.M., Cowley P.M. (2010). The use of instability to train the core musculature. Appl. Physiol. Nutr. Metab..

[28] Behm D.G., Drinkwater E.J., Willardson J.M., Cowley P.M. (2011). The role of instability rehabilitative resistance training for core musculature. Strength. Cond. J..

[29] Behm D.G., Muehlbauer T., Kibele A., Granacher U. (2015). Effects of strength training using unstable surfaces on strength, power and balance performance across the lifespan: A systematic review and meta-analysis. Sport. Med..

[30] Biscarini A., Benvenuti P., Botti F.M., Brunetti A., Brunetti O., Pettorossi V.E. (2014). Voluntary enhanced cocontraction of hamstring muscles during open kinetic chain leg extension exercise: Its potential unloading effect on the anterior cruciate ligament. Am. J. Sport. Med..

[31] Contemori S., Biscarini A. (2018). Shoulder position sense in volleyball players with infraspinatus atrophy secondary to suprascapular nerve neuropathy. Scand. J. Med. Sci. Sport..

[32] Koo T.K., Li M.Y. (2016). A guideline of selecting and reporting intraclass correlation coefficients for reliability research. J. Chiropr. Med..

[33] Hertel J., Gay M.R., Denegar C.R. (2002). Differences in postural control during single-leg stance among healthy individuals with different foot types. J. Athl. Train.

